# Spatiotemporal Processing of Bimodal Odor Lateralization in the Brain Using Electroencephalography Microstates and Source Localization

**DOI:** 10.3389/fnins.2020.620723

**Published:** 2021-01-13

**Authors:** Christine Ida Hucke, Rebekka Margret Heinen, Marlene Pacharra, Edmund Wascher, Christoph van Thriel

**Affiliations:** ^1^Department of Toxicology, Neurotoxicology and Chemosensation, Leibniz Research Centre for Working Environment and Human Factors at the TU Dortmund, Dortmund, Germany; ^2^Department Neuropsychology, Institute of Cognitive Neuroscience, Ruhr-University Bochum, Bochum, Germany; ^3^MSH Medical School Hamburg, University of Applied Sciences and Medical University, Hamburg, Germany; ^4^Department of Ergonomics, Leibniz Research Centre for Working Environment and Human Factors at the TU Dortmund, Dortmund, Germany

**Keywords:** EEG, microstates, source localization, lateralization, bimodal odors

## Abstract

The neuronal cascade related to the perception of either purely olfactory or trigeminal airborne chemicals has been investigated using electroencephalography (EEG) microstate analyses and source localization. However, most airborne chemicals are bimodal in nature, encompassing both properties. Moreover, there is an ongoing debate regarding whether there is one dominant nostril, and this could be investigated using these multichannel EEG methods. In this study, 18 right-handed, healthy participants (13 females) were monorhinally stimulated using an olfactometer with the bimodal component acetic acid during continuous EEG recording. Participants indicated the side of stimulation, the confidence in their decision, and rated the strength of the evoked perception. EEG microstate clustering determined four distinct maps and successive backfitting procedures, and source estimations revealed a network that evolved from visual-spatial processing areas to brain areas related to basic olfactory and trigeminal sensations (e.g., thalamus, cingulate cortex, insula, parahippocampal, and pre-/post-central gyri) and resulted in activation of areas involved in multisensory integration (e.g., frontal-temporal areas). Right-nostril stimulation was associated with faster microstate transition and longer involvement of the superior temporal gyrus, which was previously linked to chemical localization and provides evidence for a potential nostril dominance. The results describe for the first time the processing cascade of bimodal odor perception using microstate analyses and demonstrate its feasibility to further investigate potential nostril dominance.

## Introduction

The chemical sense, even though it is heavily intermingled, can be divided into olfaction, gustation, and trigeminal chemoreception. In this study, we focus on the perception of smells, thus focusing on olfactory and trigeminal processes. Inhaled airborne molecules can bind to olfactory receptors located in olfactory epithelium and evoke odor sensations resulting in related olfactory percepts. However, most airborne chemicals are bimodal in nature. This means that, in low concentrations, mainly the olfactory system and, in higher concentrations, additionally the trigeminal nerve endings are stimulated (Hummel and Livermore, 2002; Brand, 2006; Cometto-Muñiz and Abraham, 2016). Trigeminal stimulation evokes sensations, such as, for instance, cooling, burning, stinging, or pungent sensations, which can influence ortho- and even more retronasal odor perceptions (Laska et al., 1997; Shepherd, 2006; Shusterman, 2009; Bojanowski and Hummel, 2012). These trigeminal perceptions serve as a natural warning system that prevents potentially toxic or harmful substances from entering our bodies by evoking protective and repelling reflexes, such as sneezing (Baraniuk and Kim, 2007). In toxicology, this acute effect is related to the endpoint termed sensory irritation, which has been systematically described since the 1970s (Alarie and Stokinger, 1973). In order to estimate the individual threshold concentration for trigeminal stimulation, the airborne chemical is applied to one nostril flanked by air stimulation to the other nostril. The applied concentration is increased until a subject can determine the stimulated nostril. This process is called “lateralization” or the determination of the lateralization threshold (van Thriel et al., 2006). This lateralization paradigm takes advantage of another physiological role of the trigeminal system. Spatial orientation based on perception of airborne chemicals is a trigeminal property during bimodal odor perception (e.g., Kleemann et al., 2009). In most scenarios, humans are only able to explicitly lateralize when the trigeminal nerve is stimulated in addition to the olfactory system (Kobal et al., 1989; Cometto-Muñiz and Cain, 1998; Kleemann et al., 2009; Moessnang et al., 2011; Croy et al., 2014).

Over the last decades, the perception of airborne chemicals in humans has been increasingly investigated using a broad repertoire of neurophysiological methods. Initial challenges of investigating the neural basis of smell perception using electroencephalography (EEG) could be tackled by developing precise stimulation devices, i.e., olfactometers (Kobal and Hummel, 1988). This enabled the recording of chemosensory event-related potentials (CSERPs) mainly focusing on the N1 and P2 components. Trigeminal stimuli usually evoke faster and larger amplitudes at the vertex compared to olfactory stimulations, which are mostly slower and express lower amplitudes at more posterior electrodes (Kobal and Hummel, 1988; Rombaux et al., 2006; Iannilli et al., 2013). As mentioned before, most airborne chemicals are bimodal in nature, having olfactory and trigeminal properties. Livermore et al. (1992) explored the influence of unimodal vs. bimodal stimuli and revealed a complex interaction of the olfactory and trigeminal system as reflected by their N1–P2 components. Livermore and Hummel (2004) could demonstrate or demonstrated that bimodal stimulations evoked earlier and larger amplitudes than unimodal stimuli. Bensafi et al. (2013) reported that bimodal mixtures evoked shorter but lower N1 and P2 component amplitudes than the pure substances individually. Oleszkiewicz et al. (2019a) compared the effect of bimodal to unimodal stimulations of CSERP features and could demonstrate that CSERP amplitudes evoked by bimodal stimulation were more pronounced than unimodal stimulations separately. However, latencies were shortest for purely trigeminal stimulations with no difference in latencies between bimodal and olfactory stimulation.

Not only the stimulation devices, but also multichannel EEG recordings and related processing strategies have developed over the last decade. Topographical analysis approaches, such as microstate analysis, or source localization allow for an extraction of spatiotemporal information from multichannel EEG signals (Lehmann et al., 1987; Michel and Koenig, 2018; Michel and Brunet, 2019). Microstates are topographical patterns as measured on the scalp that remain stable for a certain duration followed by a rapid transition to the next stable topography. Each map is an outward projection of the activity of neuronal generators or a distinct network of neuronal firing (Michel and Koenig, 2018). Thus, a transition between patterns is causally linked to a change in underlying neuronal activity. By investigating and localizing (Michel and Brunet, 2019) non-random progressions of distinct microstates over time, it is possible to deduce the signal propagation in the brain reflecting successive neurocognitive processes. This technique can also be applied to study the perception of airborne chemical stimuli.

There are only few studies using such microstate and source localization analysis strategies to investigate the spatiotemporal processing of airborne chemicals. Lascano et al. (2010) investigated the differences in left- and right-sided processing of a pure odorant, hydrogen sulfide (H_2_S). Although revealing four distinctive processing steps of odor perception, a temporal as well as spatial dependency with respect to the stimulated nostril could be revealed. The authors reported an early systematic ipsilateral hemispheric processing in the primary olfactory cortex followed by a bihemispheric activation in mesial and lateral temporal and inferior frontal gyri reflecting later processing states. However, no statements regarding trigeminal processes could be made.

Thereupon, Iannilli et al. (2013) compared the neuronal activations related to the processing of the same olfactory (H_2_S) to exclusively trigeminal (CO_2_) stimulants applied to the right nostril. Trigeminal stimulation evoked faster and more pronounced CSERP components. These results were enriched by spatial information obtained from the microstate and source localization analysis. Five prominent maps were found to describe the neuronal processes estimated in the inferior, medial, and middle frontal gyrus; the middle and superior (left) temporal gyrus; cerebellum; posterior cingulate gyrus; cuneus; and the postcentral gyrus. Differences between the two stimuli types were found in the early processing stages with trigeminal activity in the posterior cingulate gyrus followed by a stronger signal for olfactory stimuli in the right ventromedial prefrontal cortex and a stronger trigeminal response in the posterior lobe of the cerebellum. Thus, it allowed for the specification of not only when, but also where processing differences between olfactory and trigeminal stimulations occur.

Thus, both modalities were investigated only separately using microstate analysis. To the authors’ knowledge, there is only one study that used bimodal odors in the context of microstate segmentation (Ohla and Lundström, 2013); however, the focus of the study on gender differences does not allow for a conclusive description of the processing cascade of bimodal odors. As mentioned before, most naturally occurring odors are bimodal in nature. Further, it is known that the two sensory trajectories do not exist in isolation, but modulate each other, which is also reflected in central processes (Brand, 2006). Iannilli et al. (2011) could show in an fMRI study that patients with selective olfactory loss who can still perceive trigeminal properties of smells (anosmia) show an altered brain response to bimodal odor stimulation compared with normosmic participants. It could be possible to investigate said modulatory effects using EEG and microstate analysis. However, closing the gap between the individual description of olfactory and trigeminal spatiotemporal processes by investigating the respective processing of bimodal stimulations would be a pre-requisite. Therefore, the first goal of this study was to describe the neuronal activation pattern of bimodal simulations using acetic acid. These can be compared to the existing microstates and source imaging studies using unimodal stimulations to determine the generalizability of certain processing cascades and brain networks and further compare these networks to fMRI findings when discussing certain areas and their potential functional roles.

Moreover, in this study, acetic acid was applied monorhinally in order to (a) ascertain that participants could clearly lateralize the stimulations as a confirmation of trigeminal involvement and (b) to compare perceptual ratings as well as the processing cascade with respect to the two nostrils. Since the early 1900s (Toulouse and Vaschide, 1900), there has been a continuous discussion of a potential nostril dominance or nostril advantage (e.g., Zatorre and Jones-Gotman, 1990; Gudziol et al., 2007; Manescu et al., 2017; Poupon et al., 2017; Zang et al., 2020). This dominance could be found on a peripheral, thus, mucosa level, or on the central level reflected in a nostril-specific brain activation pattern (Brand, 2006). With respect to a peripheral nostril dominance, the quality of the odor might be relevant. Right-nostril stimulation in an odor discrimination task led to a superior performance only if unfamiliar odors were presented (Savic and Berglund, 2000). In a PET study, Savic and Gulyas (2000) presented odors monorhinally and show that these odors were processed ipsi- and contralaterally with a general right-hemispheric dominance. Further, attempts have also been made to use EEG in order to find nostril-specific central activity patterns revealing different results when comparing CSERPs from different electrode positions evoked by left- and right-sided stimulations. Kobal et al. (1992) did find stronger responses for right-nostril stimulations. Further reports range from a contralateral (Hummel and Kobal, 1992) or one-hemispheric dominance (Olofsson et al., 2006; Rombaux et al., 2008) to no effect at all (Stuck et al., 2006). Thus, there might be a temporal and/or a spatial difference, which could not easily be characterized using conventional CSERP analyses but might be revealed by using microstate segmentation and source estimation.

Therefore, the second goal of this study is to determine if there is a nostril advance in lateralization performance, perceptual ratings, or the EEG signal in terms of microstate occurrence and respective signals’ inverse sources. If there is a difference, this approach would allow combining the temporal strength of EEG as well as the spatial power of multichannel analyses to examine when and where in the brain certain differences can be found and how that would be reflected in perceptual ratings.

## Materials and Methods

### Participants

Nineteen neurologically and psychologically healthy, right-handed participants in the age range of 18–35 years were invited to take part in the experiment. Exclusion criteria were smoking or drug use, chronic or acute airway diseases, acute allergy symptoms, or a hairstyle unsuitable for EEG recordings. Furthermore, participants were required to have a forced expiratory volume of at least 80% in a pulmonary function test (MasterScope TP, JAEGER/CareFusion, Höchberg, Germany) in order to participate in the experiment. However, as only healthy, non-smoking participants were invited, no participant needed to be excluded based on this criterion. The identification subtest of the Sniffin’ Sticks battery (Burghart Messtechnik GmbH, Wedel, Germany) served as an indicator of normal olfactory functions (Oleszkiewicz et al., 2019b).

Of the initial 19 participants, one participant did not perceive the stimulation, and thus, the experiment was aborted, and no data was collected. Thus, the final sample that entered the analysis comprised 18 participants (mean age: 26.77 years, SD age: 4.74, gender: 13 female/5 male) of which one participant only contributed half the data due to recording difficulties. However, the saved half data set of this participant is included in the final data analysis.

### Materials

#### Olfactometer

As in a previous study (Hucke et al., 2018), the stimulant acetic acid (CAS: 64-19-7, liquid concentration 30% v/v) was presented to the subject by means of a flow-olfactometer (NeuroDevice, Version 2, Warsaw, Poland). The device consists of a pump unit placed in the EEG control room, and it is connected via tubes passing through a waveguide with a repository unit in the experimental room. The pump in the control room produces a continuous, clean 2.5 l/min airstream through two default lines passing the repository unit and ending in a custom-made nose piece that comprises two separate outlets for each line, entering the left and right nostril, respectively. It allows instantly switching the flow of one of the default lines to separate tubes entering the repository unit in glass screw-capped test tubes filled with acetic acid. Thereby, the headspace (see supplement of Hucke et al., 2018, for airborne concentration) is pushed through further tubes out of the repository into the nose piece and, thus, into one of the participant’s nostrils. After 1,500 ms, the flow shifts back to the default clean air line. Thus, the olfactometer allows for a seamless and quasi-instant integration of the gaseous stimulus into the constant airstream without changing the setup of the other nostril.

Compared with the previous study (Hucke et al., 2018), the setup was optimized for EEG recordings. The repository unit of the olfactometer that includes a magnetic stirrer was covered using a custom-made steel box taped with μ-metal. Using the shield-box, the magnetic intrusion could be minimized, yet the filter and trial rejection settings during the EEG data processing were adjusted to filter potential residual magnetic influence (see section “Pre-processing”).

#### EEG System

The signal was recorded from 64 active Ag/AgCl electrodes placed in a standard 10-10 arrangement (Oosterveld and Praamstra, 2001) on an actiCap (BrainProducts, Gilching, Germany). The signal was recorded using a 1-kHz BrainAmp DC amplifier (BrainProducts, Gilching, Germany) and saved to a Windows 7 PC with the Brain Vision Recorder software (version 1.20). During the recording, FCz served as the online reference, and for display purposes, the signal was online filtered at 250 Hz. To stabilize the skin–electrode conductivity, a high-viscosity electrolyte gel was used (SuperVisc, EASYCAP GmbH, Herrsching, Germany).

### Procedure

The ethics committee of the Leibniz Research Centre for Working Environment and Human Factors at the TU Dortmund approved the study protocol. Participants were briefed about the experimental purpose and informed about the irritating sensations acetic acid might evoke and gave informed written consent. After going through the abovementioned health tests, the participants underwent an active anterior rhinomanometry (RHINO-SYS, Happersberger otopront GmbH, Hohenstein, Germany) to detect potential obstructions influencing the nasal airflow. Next, as natural nasal breathing might alter the stimulus onset and evoke respiration-triggered trigeminal stimulation by changing airflow in the nose, participants were instructed to breathe through their mouth while performing velopharyngeal closure (Kobal, 1981). After that, the EEG cap was put on the participant’s head in the dimly lit experimental room. Concurrent to the EEG recording, a functional near-infrared spectroscopy system recorded the hemodynamic response, which will be published elsewhere. The experiment was set up in PsychoPy (Peirce, 2007, 2009) and run on a Windows 10 laptop, which was connected via a wave guide to a 19” computer monitor in the experimental room.

**FIGURE 1:**
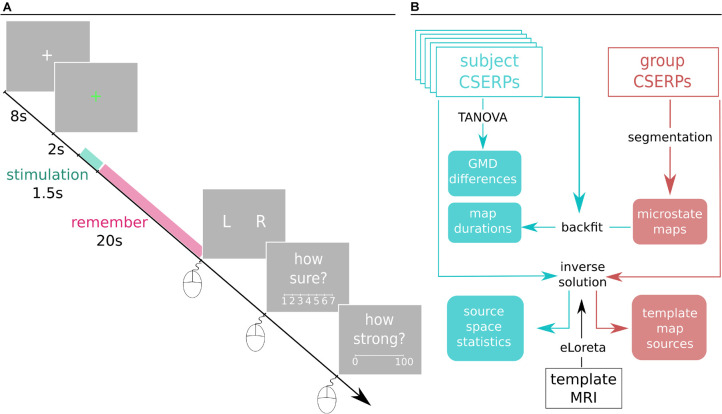
**(A)** Depiction of a trial. Each trial starts off with a white fixation cross that turns green after 8 s. After 2 s, the 1.5 s stimulation starts, and participants are instructed to detect and remember the stimulated nostril. After 20 s, participants are asked to indicate the stimulated side, rate the confidence of the nostril identification, and indicate the strength of the perceived stimulation by means of mouse clicks with the right hand. For respective scale descriptions, see main text in section “Procedure” **(B)** Spatiotemporal processing pipeline. Cyan represents subject-based CSERP data and derived results on a classical group level. Red is associated with the two grand condition averages. Black texts are processing tools, and filled boxes are results. CSEPRs: chemosensory event-related potentials, TANOVA: topographic analysis of variance, GMD: global map dissimilarity, eLORETA: exact low resolution electromagnetic tomography, MRI: magnetic resonance imaging.

The experiment consisted of 120 trials in total, 60 left- and 60 right-sided stimulations, respectively. It was divided into two blocks separated by a break. Each block was further split into sub-blocks of four random trials, after which the participant could take a short break. During the break, participants could temporarily switch to nasal breathing and drink water, preventing discomfort caused by mouth breathing. Each trial (Figure 1A) consisted of a baseline period in which a white fixation cross was displayed at the center of a gray background. After 8 s, the cross turned green, which signaled the upcoming stimulus onset in 2 s. Participants were asked to refrain from blinking after the color switch to minimize blink artifacts during the relevant time window. The participants were naïve to which nostril would be stimulated, and their task was to identify and remember the stimulated nostril. After about 20 s, a series of questions appeared on the monitor. To assure that the concentration of acetic acid in this setting indeed stimulated the trigeminal nerve, participants were asked to indicate the stimulated nostril (left, right), how certain they were of their answer (7-point Likert scale, range: “guess” to “absolutely certain”), and to rate the strength of perception on a continuous visual analog scale ranging from zero: “not perceived at all” to 100: “strongest perception imaginable” (Green et al., 1996). All answers were given using the mouse with the right hand.

After the experiment was finished, the nasal flow rate was assessed again via a second anterior rhinomanometry measurement. This allowed for excluding that one nostril swelled up and became blocked over the course of the experiment. The overall participation time was between 4 and 5 h for which participants received either 10 €/h or participation credits for their university degree.

## Analysis

### Behavioral Analysis

The behavioral analysis was executed in R (RStudio Team (2016), RStudio: Integrated Development for R. RStudio, Inc., Boston, MA^1^; version 1.1.463) using the stats package (R Core Team, version 3.5.2). A one-tailed, one-sample *t*-test was applied to test if the lateralization accuracies exceeded a chance level of 50%. Using two further one-sample *t*-tests, it was tested if the confidence and perception ratings were significantly greater than zero. Moreover, it was checked if the three behavioral parameters differed across the two experimental blocks using two-tailed paired *t*-tests. Finally, to test if left- and right-sided stimulation differed with regard to the stimulated nostril lateralization accuracy, perceptual and confidence ratings were compared for the left vs. right nostril using two-tailed, paired *t*-tests. In case of a normal distribution violation as assessed by Shapiro-Wilk tests, the corresponding nonparametric Wilcoxon signed-rank test was used.

### EEG Analysis

#### Pre-processing

The EEG signals were off-line pre-processed using MATLAB (R2018b, MathWorks, Inc., Natick, United States) EEGLAB (14.1.2b) toolbox functions. Data on the two experimental blocks were merged, electrodes were assigned to the standard 10-10 positions (Brain Electrical Source Analysis), and the signal was filtered (Hamming windowed sinc FIR band-pass filter, 0.5 Hz transition bandwidth, cutoff frequencies: 0.25–14.25 Hz, filter order: 6601). Channels with normalized kurtosis exceeding 5 SD were rejected (average of 4.61 per participant), and the signal was re-referenced to the average of the non-rejected electrodes. The continuous data was epoched from 2,000 ms preceding the stimulus onset to 6,000 ms thereafter, including a 200 ms baseline correction. Next, an iterative automatic trial rejection procedure (rejection threshold: 1,000 μV, detection prob.: 5 SD, max. % of trials rejected/iteration: 5%) was used to discard trials with artifacts. Further, an independent component analysis decomposed the signal into channel-1 components (ICs). The ADJUST (Mognon et al., 2011) and IClabel plugins (Pion-Tonachini et al., 2019) were used to detect artifact ICs, such as blinks, eye movement, or generic discontinuities, which were then removed from the data. Last, a single-equivalent current dipole model for each IC was computed by means of the spherical head model (BESA) as implemented in the DIPFIT plugin v2.3 (Oostenvelt et al., 2003). IC solutions with an estimated residual variance of more than 40% were rejected as were ICs located outside the scalp. This led to an exclusion of 14.92 ICs per participant on average. A second trial rejection procedure followed the same parameters as before. In total, the two trial rejection procedures removed an average of 23.83 trials per participant. The rejected channels were interpolated using a spherical spline interpolation.

#### Topographic Analysis

The spatiotemporal analysis was performed using the Cartool software by Denis Brunet (cartoolcommunity.unige.ch) on trials that were localized correctly and followed a recommended (Murray et al., 2008; Brunet et al., 2011; Michel and Koenig, 2018) processing pipeline (Figure 1B).

The EEG epochs were truncated (−500 to 1,700 ms), downsampled (250 Hz), and averaged with respect to the stimulus onset on the single-subject CSERPs and group CSERPs level separately for each condition. First, a nonparametric randomization test (topographic analysis of variance, TANOVA) based on the single-subject CSERPs tested for topographical differences quantified by the global map dissimilarity (GMD) between the left and right conditions (Murray et al., 2008).

Next, stable functional microstates were identified using the topographic atomize and agglomerate hierarchical clustering (T-AAHC) algorithm to segment both group CSERPs concurrently (excluding baseline) into 20 microstate maps in an incrementing iterative procedure. This means that 20 independent clustering procedures were performed with an increasing number of microstate maps. Segments that correlated more than 95% were merged. A temporal smoothing was applied (window half size of 3, strength of 10), and in addition, segments shorter than 24 ms (6 TFs) were rejected.

After selecting the optimal number of microstates based on the meta-criterion as implemented in Cartool (Bréchet et al., 2019), the resulting prototype maps were fitted back to the group CSERPs (excluding baseline) by assigning the prototype map with the highest spatial correlation to each time point, taking signal polarity into account. Again, temporal smoothing and a small segment rejection were applied. Based on this backfit, a specific time window was visually determined in which the topography of the two conditions potentially differ. To test this difference statistically, the templates were fitted to the single-subject CSERPs (same parameters) in this selected time window, and the mean durations of each map within the specified time range were extracted. This duration of selected maps, which were present in the group CSERP backfits in the window of interest, were exported and analyzed using R [stats package, R Core Team, version 3.5.2; plyr (Wickham, 2011); ggplot2 (Wickham, 2016)]. The data was checked for a normal distribution of map duration differences using the Shapiro-Wilk test and compared using either a paired *t*-test or the nonparametric Wilcoxon signed-rank test. When applicable, *p*-values were Bonferroni-adjusted to control for multiple comparisons.

#### Source Estimation

The sources were estimated as described by Michel and Brunet (2019). First, the EEG positions were co-registered to the MNI 152 head template (retrieved on 24.03.2020)^2^ as implemented in the toolbox from which a gray matter mask was extracted, serving as a solution space for 5,000 symmetrical solution points. Based thereupon, the locally spherical model with anatomical constraints (LSMAC) (Brunet et al., 2011) was used to calculate the lead field, incorporating the mean participant age. This forward solution was inverted, thus solving the inverse problem using the exact low resolution electromagnetic tomography (eLORETA) algorithm (Pascual-Marqui, 2007; Pascual-Marqui et al., 2011). To determine the sources of the microstate maps in the EEG signal, the resulting inverse solution was multiplied with the group CSERPs. For each condition, the time window of the maps fitted on the group CSERPs was averaged and localized in the inverse space.

Finally, the inverse solution was multiplied with the condition-specific, single-subject CSERPs for which FDR-corrected paired *t*-tests (thresholding *p*-value at 0.01) determined the time and area within the brain where the two conditions differed. The resulting *t*-map was exported for visualization purposes to MRIcroGL.

## Results

### Behavioral Results

Participants were able to correctly lateralize the stimuli (*M* = 93.47%, *SD* = 5.71, median = 95.83%, IQR = 86.88–97.50%), which significantly exceeded chance levels, *Z* = 3.71, *p* < 0.001, effect size *r* = 0.87. Participants were confident in their ratings as evident by a significantly higher (range: 1–7: *M* = 5.35, *SD* = 0.94) than zero rating, *t*(17) = 24.13, *p* < 0.001. Participants also rated their perception (transformed range 0–100: *M* = 44.98, *SD* = 19.58) as being significantly stronger than “not perceived at all,” *t*(17) = 9.75, *p* < 0.001. Participants did not lateralize [*t*(17) = −0.88, *p* = 0.39], rate confidence [*t*(17) = −1.48, *p* = 0.16] or perceptions [*t*(17) = −0.97, *p* = 0.35] any differently across the two experimental blocks.

With regard to nostril differences, the lateralization accuracy (*Z* = −0.28, *p* = 0.78, effect size *r* = 0.07) and confidence [*t*(17) = −0.78, *p* = 0.45] did not differ. The perceptual ratings of left (*M* = 41.40, *SEM* = 4.53) vs. right (*M* = 48.56, *SEM* = 5.30) nostril stimulation differed marginally although not significantly, *t*(17) = −2.06, *p* = 0.06.

### Topographical Results

**FIGURE 2:**
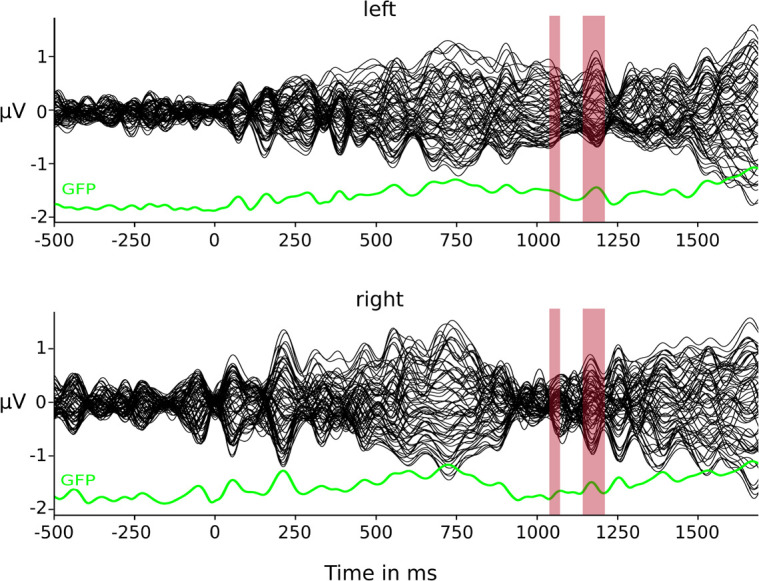
Butterfly plots of group CSERP data. Condition-specific group averages for the left- and right-sided conditions. Each line represents one electrode. In green depicted below each group CSERP is the respective global field power (GFP). Red highlights represent time window of significant GMD differences.

**FIGURE 3:**
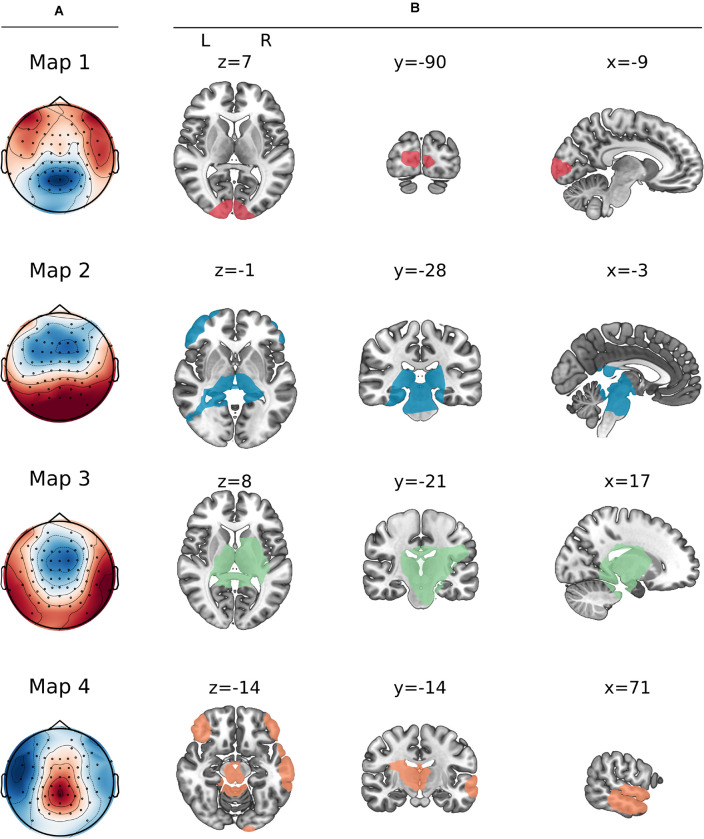
Prototype maps resulting from the clustering step. **(A)** Map topographies of the four prototype maps. **(B)** Source localization of the maps. X, Y, and Z correspond to coordinates in MNI space.

The TANOVA on GMD between the two conditions (Figure 2) resulted in two time windows of statistically significant differences: 1,048–1,068 and 1,144–1,204 ms post-stimulus onset. The clustering method and resulting meta-criterion determined the optimal number of four prototype microstate maps (Figure 3A) explaining 85.3% of global variance.

**FIGURE 4:**
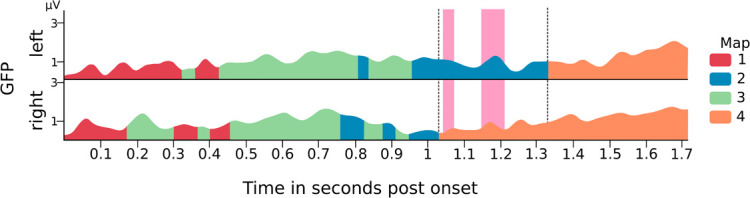
Backfit of the prototype maps on the group CSERPs global field power (GFP) of each condition with fitted maps. Pink marks highlight significant time intervals of the TANOVA results on GMD. Dotted black line encloses the time window used based thereupon for the subject-level backfit.

The backfits of these four prototype maps to the group CSERPs revealed no condition-specific maps. Yet there seemed to be a 300 ms time window (1,032–1,328 ms) in which the two conditions differed with map 2 still being present in left-sided stimulation and map 4 being more dominant in the right-sided condition (Figure 4).

**FIGURE 5:**
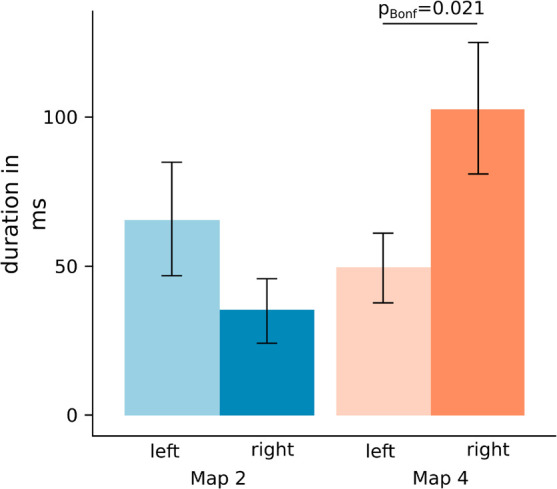
Mean map duration of map 2 (blue) and map 4 (orange) resulting from the backfit on subject CSERPs. Map duration for map 2 did not but for map 4 did differ significantly between left- and right-sided stimulation. Error bars represent SEM. pBonf: Bonferroni corrected *p*-value.

This time window enclosed the significant GMD differences and was, thus, used as the outer bounds for the backfit on the subject level to extract the map duration of maps 2 and 4 (Figure 4). As the Shapiro-Wilk test for normality indicated a non-normal distribution of the paired differences (*p* = 0.012) a Wilcoxon approximation signed-rank test was applied. As can be seen in Figure 5, the duration of map 2 after left (median = 46.80 ms, IQR = 29.37–67.00 ms) and right-sided (median = 16.00 ms, IQR = 0–56.64 ms) stimulation was not significantly different. However, the comparison of map 4 duration between the left (median = 51.91 ms, IQR = 28.94–59.87 ms) and right-nostril (median = 79.86 ms, IQR = 42.46–126.20 ms) stimulation showed a significant difference, *Z* = −2.56, *pBonf* = 0.02, effect size *r* = 0.60.

### Source Estimation Results

#### Prototype Maps

The neuronal processing cascade of involved networks underlying the topographical maps was determined by applying the inverse solution to the group CSERPs (Figure 3B and Table 1).

**TABLE 1:** Estimated sources associated with the four prototype maps and *t*-test results. L/R correspond to the left and right hemispheres.

Map 1		Map 3		Map 2		Map 4		*t*-test	
L	R	L	R	L	R	L	R	L	R
				Cerebellum	Cerebellum	Cerebellum	Cerebellum	Cerebellum	
Cuneus	Cuneus						Cuneus	Cuneus	Cuneus
						Pre-cuneus	Pre-cuneus	Pre-cuneus	Pre-cuneus
Middle occipital gyrus				Middle occipital gyrus					
		Thalamus	Thalamus	Thalamus	Thalamus	Thalamus	Thalamus	Thalamus	Thalamus
		Cingulate cortex	Cingulate cortex	Cingulate cortex	Cingulate cortex	Cingulate cortex	Cingulate cortex	Cingulate cortex	Cingulate cortex
		Brainstem	Brainstem	Brainstem	Brainstem			Brainstem	
		Parahipp. gyrus	Parahipp. gyrus	Parahipp. Gyrus	Parahipp. gyrus	Parahipp. gyrus	Parahipp. gyrus	Parahipp. gyrus	Parahipp. gyrus
			Insula			Insula			
			Putamen						
			Pre-central gyrus					Pre-central gyrus	Pre-central gyrus
			Post-central gyrus						Post-central gyrus
			Inferior parietal cortex						
				Inferior temporal gyrus			Inferior temporal gyrus	Inferior temporal gyrus	
				Middle temporal gyrus			Middle temporal gyrus	Middle temporal gyrus	Middle temporal gyrus
							superior temporal gyrus		
				Fusiform gyrus					
				Inferior frontal gyrus	Middle frontal gyrus	Inferior frontal gyrus	Inferior frontal gyrus		
				Middle frontal gyrus	Inferior frontal gyrus	Middle frontal gyrus	Middle frontal gyrus		
				Superior frontal gyrus					
									Superior parietal gyrus
									Angular gyrus
									Uncus

*Note that the map order corresponds to the respective backfit: maps 1–4.*

Map 1, present as the first map in both conditions, was localized in the cuneus. This was followed by map 3, which originated in a larger network, including the brainstem and thalamus, the parahippocampal gyrus, (posterior) cingulate gyrus, and a right hemispheric cluster ranging from the insula and putamen to the pre- and post-central gyrus and a cluster in the neighboring inferior parietal lobe. Next, map 2 included overlapping areas, such as the brainstem, thalamus, and parahippocampal gyrus, but further included the cerebellum with a small cluster ranging into the left middle occipital gyrus. Moreover, a temporal cluster emerged covering parts of the left inferior, middle, and fusiform gyrus. A further cluster was localized in the left frontal region ranging from the inferior over the middle to the superior frontal gyrus. A weaker counterpart was found in the right hemisphere only, including the middle and inferior frontal gyrus. Map 4 was also found to have neuronal generators in the cerebellum, thalamus, posterior cingulate, and parahippocampal gyrus. Additionally, the frontal regions were included as well, yet expressing a stronger symmetry across the hemispheres. Next to the overlapping regions, map 4 had a unique cluster in the pre-cuneus and further partly shared sources with maps 1 (right cuneus) and 3 (left insula). An additional large cluster was found in the right inferior, middle, and superior temporal gyrus.

#### Statistics in Source Space

**FIGURE 6:**
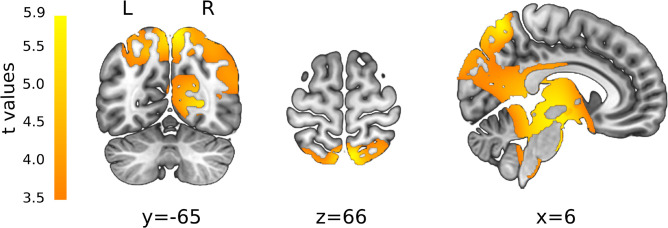
*t*-maps resulting from statistical comparison between the left- and right-sided stimulation (L > R). All *t*-values were positive, representing a stronger associated activity for left-sided stimulation. Color gradient from orange to yellow is associated with increasing *t*-values. L, left; R, right orientation. X, Y, and Z are coordinates in MNI space.

After convolving the single-subject CSERPs with the inverse matrix, the FDR-corrected, paired *t*-test revealed significant time (1,012–1,028 ms) and solution points in the inverse space (Figure 6). All areas were significantly more associated with left-sided stimulation and included the cerebellum, (pre)cuneus, thalamus, cingulate gyrus, brainstem, inferior and middle temporal gyrus, pre- and post-central gyrus, superior parietal, and angular gyrus or uncus. When testing the reversed contrast (right > left nostril), no additional significant results were found other than negative *t*-values in the same areas reported for the first contrast.

## Discussion

### Microstate Maps and Sources for Bimodal Odor Stimulation

The first aim of this study was to describe and disentangle the central nervous processing cascade of bimodal odors and to compare the respective results to those of Lascano et al. (2010), who used a pure olfactory stimulus, and Iannilli et al. (2013), who compared olfactory to trigeminal stimulations. The question was in how far the neuronal processing steps of the distinct networks overlap. The EEG microstate clustering procedure of the two condition group CSERPs revealed 4 maps to be the optimal number for the current experiment. Fitting the prototype maps back to the condition group CSERPs, the same temporal map progression in both conditions could be revealed: map 1, map 3, and map 2 followed by map 4.

Map 1 was characterized by a posterior negativity and a lateral-anterior positivity. This map was localized in the occipital gyrus and more specifically in the cuneus and left middle occipital gyrus. The cuneus is commonly associated with basic visual feature processing, in this case potentially elicited by the fixation cross before the first chemical molecules actually bind to the receptors. However, the EEG microstates study by Iannilli et al. (2013) also found the cuneus to be associated with early trigeminal processing. Further, fMRI studies found significant cuneus activity linked to the processing of chemical stimuli (e.g., Albrecht et al., 2010; Billot et al., 2011). In an fMRI study by Porter et al. (2005) participants were instructed to either identify or localize chemical stimulations. Different task instructions activated specific task-related areas with the cuneus being one of the key areas related to identification of the chemical stimulus. Thus, the initial activation in this study or also in the study by Iannilli et al. (2013) might be associated with the detection or identification of a stimulus as a pre-requisite step of localizing it. The middle occipital gyrus was previously shown in fMRI studies to be involved in spatial processing (Cona and Scarpazza, 2019), even being preserved in the blind population (Renier et al., 2010). Thus, the initial occipital involvement might reflect a spatial visualization component linked to the detection but potentially also the development of a spatial representation of the chemical stimulus in the immediate environment.

Next, map 3 superseded map 1 at 150–300 ms after stimulus onset lasting until 750–950 ms and was dominated by a fronto-central negativity and lateral-temporal positivity. Its sources were estimated to be areas previously associated with the processing of chemical stimuli in fMRI studies: bihemispheric activation in the brainstem, thalamus, and parahippocampal gyrus and right hemispheric activity in the cingulate cortex, insula, putamen, pre- and post-central gyri (Albrecht et al., 2010), and inferior parietal lobule. Map 3 microstate sources from the insula and the parahippocampal gyrus are in line with sources reported by Lascano et al. (2010), and they are involved in olfactory processes. Iannilli et al. (2013) associated the cingulate cortex specifically with trigeminal stimulations. A map occurring later in the same study was estimated to arise from, among other areas, the postcentral gyrus, which is linked to the detection and evaluation of sensory features of trigeminal stimuli (Lundström et al., 2011). A meta-analysis of functional MRI studies on chemosensation (Albrecht et al., 2010) revealed an involvement of the insula, the cingulate cortex and the primary and secondary somatosensory cortices in the context of trigeminally evoked nociception. Thus, following the initial step of developing a representational environment (map 1), map 3 might reflect more specifically initial olfactory as well as trigeminal processing stages.

The topography of map 2, present at the earliest from 750 to 800 ms, was dominated by an anterior negativity and posterior positivity. Map 2 was localized in partly overlapping areas found for the preceding map 3 (brainstem, thalamus, cingulate cortex, and parahippocampal gyrus). Further, sources were estimated in the cerebellum, left inferior, and middle temporal regions as well as bilateral, yet left-dominating frontal regions. Map 4 was localized in the same overlapping areas as maps 2 and 3. In contrast to map 2, map 4 showed a balanced bihemispheric frontal pattern and right-hemispheric temporal involvement, further including the superior temporal gyrus and the insula. The frontal and temporal areas were previously reported to be involved in olfactory microstate sources as described by Lascano et al. (2010) and in olfactory as well as trigeminal microstate sources reported by Iannilli et al. (2013). In addition to this, Iannilli et al. (2013) found the cerebellum to be associated with the processing of olfactory stimuli. The role of the cerebellum in the perception of trigeminal stimuli was also highlighted in the aforementioned meta-analysis by Albrecht et al. (2010). Sources of maps 2 and 4 are not only related to olfactory and trigeminal processes, but further play a vital role in multisensory integration. The inferior middle frontal gyri and the superior temporal gyrus are part of an association network, integrating multisensory stimuli (Boyle et al., 2007a; Albrecht et al., 2010). These findings were further supported by an fMRI study by Pellegrino et al. (2017). In their study, participants were presented with either unimodal or bimodal chemosensory stimuli. Compared to unimodal stimulation, bimodal stimuli elicited activation of the insula, cerebellum, cingulate cortex, and superior frontal gyrus, validating their role in the integration of multisensory stimuli with both olfactory and trigeminal properties. Additionally, our findings might be in line with the suggestion by Pellegrino et al. (2017) that the thalamus modulates attention to the two stimulus modalities. Moreover, the superior temporal gyrus was reported to be a key area in the aforementioned fMRI study by Porter et al. (2005), where it was found to be involved in the localizing as contrasted to identifying chemical stimuli. Therefore, this area might be associated with a potential marker of a nostril-dependent activation of this study, discussed in the next section.

In conclusion, we identified an evolving network of brain areas involved in the temporal processing of bimodal odor perception. Areas that were previously linked to either unimodal stimulation or multisensory integration can now be shown for the first time in a temporal framework for mixed olfactory-trigeminal stimulation: Starting with the development of a spatial representation of the stimulus, continuing with more specific trigeminal and olfactory feature processing, and ending in a higher order multisensory integration of the bimodal stimulus. This result closes the gap of the olfactory and trigeminal cascades studied in isolation (Lascano et al., 2010; Iannilli et al., 2013). The revealed cascade can now serve as a starting point to further study modulations of the two trajectories. Future studies can compare temporal effects of sensory modulation as reported by Iannilli et al. (2011) using a pure olfactory, trigeminal, and bimodal odor in a healthy compared to an anosmic population.

### Left vs. Right Nostril Differences: Is There a Nostril Dominance?

Comparing the microstate map durations with respect to nostril side, right-nostril stimulation evoked a longer presence of map 4. This difference is, in itself, no “advantage” for the right nostril *per se*. In order to argue for any sort of advantage, we would need a behavioral endpoint to put this map duration difference into perspective. Map 4 sources include the superior temporal gyrus, which was previously shown to be associated with chemical localization tasks (Porter et al., 2005; Frasnelli et al., 2012). However, no localization performance difference could be shown as a function of nostril side. This result might be due to a localization performance clearly above chance in this particular participant sample. Thus, localization performance might not have been a suitable measure of nostril-dominance.

Alternatively, perceptual ratings might offer a more sensible endpoint in the current setting. Indeed, a tendency for higher right-nostril perceptual strength was found in this study. The authors are aware that the statistical cutoff of 0.05 was not met in the direct comparison (*p* = 0.06), yet interesting follow-up questions could be generated, nonetheless. Therefore, a short discussion of the perceptual ratings is included, which indicates a tendency for higher right-nostril perceptual strength. In this case, the superior temporal gyrus might not only be involved in localization, but also in perceptual evaluation. One might argue that the localization decision of where a stimulation occurred is based on the molecule concentration, which is, in fact, similar to sound loudness in auditory perception, a physical determinant of perceptual intensity (e.g., Frasnelli et al., 2003). Therefore, the lateralization task is needed to infer nostril-specific differences on a neural level, yet the most useful behavioral measure is the perceptual rating. This rating is indicated on a continuous scale as opposed to a dichotomous left–right decision. However, future studies are needed to test the hypothesis that the superior temporal gyrus is not only involved in the lateralization task but also in perceptual evaluation of the stimulation side as, thus far, only other regions have been associated with intensity coding (Bensafi et al., 2008). Moreover, Oertel et al. (2012) report the superior temporal gyrus to be associated with the coding of physical intensity of the stimulus irrespective of the reported pain perception evoked by high concentrations of CO_2_. It is possible that these irritating perceptions are the fundamental tool to come to a side-conclusion. The assumption could be investigated by contrasting trials with either localization or perceptual rating instructions. This could answer the question of when (during lateralization and evaluation) and where (superior temporal gyrus) a nostril dominance exists. Further down the line, this might help in understanding how humans are able to navigate based on their chemical sense. Trigeminal perceptions seem to be the basis of localizing odors, and thus, the superior temporal gyrus might be involved in this process as well.

The statistics in the inverse space reveal significantly stronger activity after left-nostril stimulation at one time window around 1 s post-stimulus, right before the map switch from map 2 to 4 in the right-nostril condition. Conversely, at this time point, underlying processes associated with map 2 were not yet completed after left-nostril stimulations. Thus, sources related to map 2 were unsurprisingly among the significant areas, e.g., the brainstem, the cerebellum, and the left inferior temporal gyrus. These regions are partly in line with findings by Boyle et al. (2007b), who also contrasted left- vs. right-nostril stimulation. They found higher activations of the brainstem and the cerebellum for left-nostril stimulation; areas found in the current study for the same contrast. In addition to these regions, Boyle et al. (2007b) found the superior temporal gyrus to be more activated in left-nostril trials though the current study could link this area to map 4, which was more representative of right-nostril stimulation as discussed previously. Next, the pre- and post-central gyri were linked to map 3 and were mainly associated with lower-level feature processing of bimodal stimulations. It might be possible that left-nostril processing was overall slower and took longer in this initial low-level somatosensory stage. Meanwhile right-nostril processes related to this stage were already completed and on the verge of progressing to the higher-level multisensory integration stages. Therefore, one might argue for a right-nostril dominance in terms of processing speed and faster progression to the final stage associated with map 4. Last, the superior parietal lobule, the angular gyrus, or the uncus were not found to be associated with any prototype map source. Though not linked to microstate sources, these regions can be associated with chemosensory processes. Whereas the uncus is part of the primary olfactory cortex (Sobel et al., 2000), the aforementioned fMRI meta-analysis by Albrecht et al. (2010) identified the superior parietal lobule to be involved in trigeminal stimuli processing. Higher activations of the angular gyrus for the left-nostril condition could be related to its role in the processing of olfactory stimulus intensities as found by Bensafi et al. (2008), thus further supporting the idea of a prolonged basic olfactory and trigeminal processing stage in left-nostril stimulation. Overall, the results of the inverse statistic do not exclusively reflect stages represented by the microstate prototype sources. This highlights the difference between the group CSERPs prototype sources representing the more general activation pattern after bimodal stimulation and the inverse statistics based on the single subject CSERPs data.

### Limitations

Microstates are clustered based on grand averages, which, therefore, might lose in precision because not all maps and respective sources need to be present or involved in each individual as has been previously discussed by Iannilli et al. (2013). Furthermore, the group CSERPs backfit, which guides the hypothesis generation of which time windows might be of interest and directs the statistical analysis, is based on a winner-take-all approach. More technically, the relatively low airflow of the olfactometer could have reduced the temporal precision. This might have affected the precise map onsets in the overall average. However, it was still possible to determine reasonable microstate maps based on the current study setup. For follow-up studies, it might be an option to use an olfactometer with a higher airflow, especially if more specific temporal hypotheses are tested. Yet, based on the current results, the airflow used here was sufficient for an initial assessment and a more explorative approach that is linked to the results of previous studies. Next, the microstate results interpretation highly depends on the chosen number of microstates. The selected number of four out of up to 20 microstates was based on the meta-criterion as available in Cartool, integrating several decision criteria (GEV, cross-validation, KL, etc.). The lack of previous experiments prevented an *a priori* decision on how many microstates to use. Using four microstates could only reveal a rather crude network associated with each map. Choosing a higher number of microstates might enable the identification of more distinct areas and respective temporal precision of the more global network shown here. However, Lascano et al. (2010) and Iannilli et al. (2013) also found four maps for each stimulus type to be the best representing number of microstate maps. Another limiting factor concerning the inverse approach is the lack of individual structural MRI images and actual electrode positions of each subject. The inverse solution was based on a template image and standard electrode position coordinates. This affects the spatial precision. Regions found here were still merged into broader networks as an inverse estimation does not offer the same spatial resolution and precision as, for instance, a high-field fMRI, especially in deeper structures related to primary olfactory processes. These factors could be optimized in future studies. Future studies elaborating on nostril dominance might also include a left-handed population as well as investigate the effect of ocular or auditory dominance. This could reveal an overall tendency of population-based sensory dominance and its interaction with handedness.

## Conclusion

We identified a network involved in the processing of bimodal odors consisting of several stages, coherent with previous studies focusing on unimodal stimulations. This network evolves from visual-spatial processing areas to brain areas related to basic olfactory and trigeminal sensations and results in activation of temporal and frontal areas involved in multisensory integration. Brain areas found during later stages were associated with task-related processes and showed significant differences during left- vs. right-nostril stimulation. Right-nostril stimulation was associated with faster microstate transition and longer involvement of the superior temporal gyrus, which was previously linked to chemical localization. These findings on the neural level might reflect a right-nostril dominance with faster processing of right-sided stimuli though no nostril advantage, except for a tendency for stronger right-nostril perception, could be found on the behavioral level. This study further demonstrates the feasibility of multichannel EEG approaches to study chemosensory-related processes and validates the method as a tool for future studies to further investigate potential nostril dominances.

## Data Availability Statement

The datasets presented in this study can be found in online repositories. The names of the repository/repositories and accession number(s) can be found below: OSF repository (https://osf.io/w9zvq/).

## Ethics Statement

The studies involving human participants were reviewed and approved by the Ethics Committee of the Leibniz Research Centre for Working Environment and Human Factors at the TU Dortmund. The patients/participants provided their written informed consent to participate in this study.

## Author Contributions

CH: conceptualization, methodology, validation, formal analysis, investigation, data curation, writing—original draft, writing—review and editing, visualization, and project administration. RH: validation, formal analysis, data curation, writing—original draft, writing—review and editing, and visualization. MP: conceptualization and writing—review and editing. EW: resources and writing—review and editing. CvT: conceptualization, methodology, resources, writing—review and editing, supervision, and project administration. All authors contributed to the article and approved the submitted version.

## Conflict of Interest

The authors declare that the research was conducted in the absence of any commercial or financial relationships that could be construed as a potential conflict of interest.

## Footnotes

1 http://www.rstudio.com/

2 https://sites.google.com/site/cartoolcommunity/files
